# Automated quantification of vacuole fusion and lipophagy in *Saccharomyces cerevisiae* from fluorescence and cryo-soft X-ray microscopy data using deep learning

**DOI:** 10.1080/15548627.2023.2270378

**Published:** 2023-10-31

**Authors:** Jacob Marcus Egebjerg, Maria Szomek, Katja Thaysen, Alice Dupont Juhl, Suzana Kozakijevic, Stephan Werner, Christoph Pratsch, Gerd Schneider, Sergey Kapishnikov, Axel Ekman, Richard Röttger, Daniel Wüstner

**Affiliations:** a Department of Biochemistry and Molecular Biology, University of Southern Denmark, Odense M, Denmark; b Department of Mathematics and Computer Science, University of Southern Denmark, Odense M, Denmark; c Department of X‑Ray Microscopy, Helmholtz-Zentrum Berlin, Germany and Humboldt-Universität zu Berlin, Institut für Physik, Berlin, Germany; d SiriusXT, 9A Holly Ave. Stillorgan Industrial Park, Blackrock, Co, Dublin, Ireland; e Department of Biological and Environmental Science and Nanoscience Centre, University of Jyväskylä, Jyväskylä, Finland

**Keywords:** Deep learning, lipophagy, Niemann-Pick disease, segmentation, tomography, X-ray

## Abstract

During starvation in the yeast *Saccharomyces cerevisiae* vacuolar vesicles fuse and lipid droplets (LDs) can become internalized into the vacuole in an autophagic process named lipophagy. There is a lack of tools to quantitatively assess starvation-induced vacuole fusion and lipophagy in intact cells with high resolution and throughput. Here, we combine soft X-ray tomography (SXT) with fluorescence microscopy and use a deep-learning computational approach to visualize and quantify these processes in yeast. We focus on yeast homologs of mammalian NPC1 (NPC intracellular cholesterol transporter 1; Ncr1 in yeast) and NPC2 proteins, whose dysfunction leads to Niemann Pick type C (NPC) disease in humans. We developed a convolutional neural network (CNN) model which classifies fully fused versus partially fused vacuoles based on fluorescence images of stained cells. This CNN, named Deep Yeast Fusion Network (DYFNet), revealed that cells lacking Ncr1 (*ncr1∆* cells) or Npc2 (*npc2∆* cells) have a reduced capacity for vacuole fusion. Using a second CNN model, we implemented a pipeline named LipoSeg to perform automated instance segmentation of LDs and vacuoles from high-resolution reconstructions of X-ray tomograms. From that, we obtained 3D renderings of LDs inside and outside of the vacuole in a fully automated manner and additionally measured droplet volume, number, and distribution. We find that *ncr1∆* and *npc2∆* cells could ingest LDs into vacuoles normally but showed compromised degradation of LDs and accumulation of lipid vesicles inside vacuoles. Our new method is versatile and allows for analysis of vacuole fusion, droplet size and lipophagy in intact cells.

**Abbreviations:** BODIPY493/503: 4,4-difluoro-1,3,5,7,8-pentamethyl-4-bora-3a,4a-diaza-*s*-Indacene; BPS: bathophenanthrolinedisulfonic acid disodium salt hydrate; CNN: convolutional neural network; DHE; dehydroergosterol; *npc2∆*, yeast deficient in Npc2; DSC, Dice similarity coefficient; EM, electron microscopy; EVs, extracellular vesicles; FIB-SEM, focused ion beam milling-scanning electron microscopy; FM 4-64, *N*-(3-triethylammoniumpropyl)-4-(6-[4-{diethylamino} phenyl] hexatrienyl)-pyridinium dibromide; LDs, lipid droplets; Ncr1, yeast homolog of human NPC1 protein; *ncr1∆*, yeast deficient in Ncr1; NPC, Niemann Pick type C; NPC2, Niemann Pick type C homolog; OD_600_, optical density at 600 nm; ReLU, rectifier linear unit; PPV, positive predictive value; NPV, negative predictive value; MCC, Matthews correlation coefficient; SXT, soft X-ray tomography; UV, ultraviolet; YPD, yeast extract peptone dextrose

## Introduction

Lipids are important for the structural integrity of cellular membranes and also function as regulators of membrane fusion and fission events. Beside membrane lipids, cells store triacyl esters of glycerol and fatty acyl esters of sterols as neutral lipids in lipid droplets (LDs). Lipophagy is an autophagic process that involves the degradation of LDs within degradative compartments, such as lysosomes in mammalian cells or its equivalent, the vacuole, in yeast cells. Lipophagy allows cells to clear excess lipids, which prevents lipid accumulation, playing an important role in maintaining cellular energy homeostasis and regulating lipid metabolism. Lipophagy is gaining interest as an important cellular pathway, not only for mobilizing stored lipids and providing precursors for membrane synthesis, but also as potential mechanism for clearance of misfolded or aggregated proteins, which have been shown to accumulate on the droplet surface [1,2]. Defective lipid trafficking is a hallmark of many neurodegenerative diseases, including Niemann Pick type C (NPC) disease. In this lipid storage disorder mutations in either NPC1 or NPC2 proteins lead to accumulation of cholesterol and sphingolipids, such as sphingosine, glycosylceramide and sphingomyelin, in lysosomes [3,4]. Mammalian NPC2 has been proposed to pick up cholesterol derived from low density lipoprotein and other sources inside lysosomes and deliver it to NPC1 for integration into the lysosomal membrane [5,6]. Recently, both proteins have been implicated in autophagy in yeast, as normal vacuole function and lipid mobilization from LDs were compromised in cells lacking functional Ncr1, the yeast homolog of NPC1, or yeast Npc2 during starvation [7,8]. Yeast vacuoles retain many functions of mammalian lysosomes, and they are due to their larger size amenable to microscopic observation. Extensive fusion and fission of vesicles maintain a steady state of vacuolar size, and vacuole vesicles are known to fuse to one central compartment during starvation [9,10]. Analysis of vacuole fusion, both in cells and in reconstituted systems, has contributed significantly to our understanding of the membrane fusion machinery, which is conserved from yeast to humans [9,11]. In addition, the same regulatory lipids and nutrient-dependent signaling pathways have been shown to control the morphology and function of the yeast vacuole and of mammalian lysosomes [9,10]. These similarities, together with the much simpler genetic manipulation compared to mammalian cells make yeast cells a widely established model for studying homotypic membrane fusion, lipophagy and other lysosome-related processes [2,9,10,12]. Also, Ncr1 and Npc2 show high structural similarity to mammalian NPC proteins, and both, NPC1- and NPC2-deficient mammalian cells can be rescued by the *S. cerevisiae* counterpart [13,14].

Vacuole morphology is often assessed by manual scoring and/or by counting of vacuolar vesicles attached to each other [7,8,11,15–17]. While this is a valid procedure, it is time-consuming and difficult to standardize between labs and individuals. Similarly, the extent of lipophagy is often assessed by manual counting of LDs stained with dyes, such as BODIPY 493/503, inside the vacuole co-labeled with complementary dyes, such as FM 4–64 [18–20] or just based on bright field images of the vacuole [7]. The number of ingested LDs is typically assessed in epifluorescence or confocal images, a tedious procedure, which suffers from the limited resolution of conventional fluorescence microscopy which at best allows one to discern cellular structures being about 300 nm apart. Furthermore, structural changes in vacuoles or accumulation of other lipid assemblies inside vacuoles apart from LDs might be missed by this approach. To improve the resolution, various approaches have been used; e.g., single-molecule localization microscopy of BODIPY dyes or photoactivatable fluorescent proteins can provide information about droplet localization inside and outside of vacuoles with a resolution of 30–50 nm [21,22]. This approach, however, does not alleviate the need for staining, thereby largely missing the cellular context in which lipophagy and other autophagic processes take place. Alternatively, electron microscopy (EM) can provide ultrastructural information of vacuoles and LDs with < 10-nm resolution and in a label-free manner but only for thin slices due to the low penetration depth of electrons in biological samples [18]. Freeze-etch EM is based on fracturing frozen specimen, thereby obtaining a replica of the original biological structure [23]. This method has been used to determine the ultrastructure of vacuoles in wild-type and Ncr1- and/or Npc2-deficient yeast cells [7]. Depending on the used freezing technique and fracture protocol, membrane deformation and structural rearrangements cannot be excluded in this technique [24]. The best possible resolution is currently obtained with focused ion beam milling scanning electron microscopy (FIB-SEM), allowing for attaining close-to molecular resolution in three dimensions. This comes to the price of slow acquisition speed and need for proper fixation and embedding of the specimen with very long acquisition times of many hours up to several days per cell [25]. Soft X-ray tomography (SXT) achieves 3D information with isotropic 30-nm resolution of intact cryo-frozen cells throughout their entire thickness without the need for sample embedding or fixation [26,27]. Its contrast is based on the extent by which organelles absorb X-rays which is high for lipid membranes and LDs and low for the cytosol or water-filled vacuole, allowing for ultrastructural analysis of cells including starvation responses in yeast [28–31]. SXT has an about 10-fold higher resolution than diffraction-limited fluorescence microscopy but about 5-fold lower resolution compared to FIB-SEM. However, SXT is much faster than FIB-SEM as it allows for 3D imaging of entire cells in their near-native state in about 30–45 min, depending on the used photon dose. Also, SXT is not invasive and allows for assessing cellular ultrastructure not only in selected slices but in the entire 3D volume of a cell, in contrast to classical EM techniques. SXT relies on the energy range known as the *water window*, which is defined as the X-ray absorption of the *K* edges of oxygen (543 eV, 2.28 nm) and carbon (284 eV, 4.37 nm) [32]. By working within this energy window there is no need for additionally staining of biological samples, hence a natural absorption contrast will emerge from the biological carbon-rich structures. SXT is therefore ideally suited to study cellular ultrastructure at the mesoscale, bridging the gap between electron and light microscopy. SXT can be combined with cryo fluorescence microscopy, allowing for subcellular imaging and phenotype mapping with molecular specificity and over entire cell volumes. A bottleneck in the analysis of 3D image stacks reconstructed from X-ray tomograms is the lack of suitable software tools for organelle segmentation, as only very few studies have attempted to use modern machine learning approaches to segment X-ray images in an automated manner for selected problems [33,34]. This leaves the segmentation task often to tedious manual annotation of images in specialized settings and research environments.

In this study, we employ a combination of SXT and fluorescence microscopy and use deep learning to quantify the processes of vacuole fusion and lipophagy in yeast cells. To accomplish this, we have created two convolutional neural network (CNN) models; the first model, which we named DYFNet (Deep Yeast Fusion Network), is used for automated classification of yeast vacuole shapes according to their fusion state. This allows for automatic scoring of fully and partially fused vacuoles and thereby to determine the role of Ncr1 and Npc2 in vacuole fusion. The second CNN model is part of a pipeline called LipoSeg which allows for automated segmentation of X-ray images and thereby for measuring the ingestion of LDs into vacuoles of both wild-type yeast and yeast cells lacking either Ncr1 (*ncr1∆* cells) or Npc2 (*npc2∆* cells) from X-ray tomograms. By this computational approach we are able to create fully automated 3D renderings of LDs inside and outside of the vacuole to measure droplet volume, number, and distribution. We discovered that yeast cells lacking either Ncr1 or Npc2 have partially fragmented vacuoles and a compromised ability to mobilize lipids from LDs, despite efficient droplet uptake into the vacuole. Our novel combination of automated phenotype classification and instance segmentation based on fluorescence and X-ray microscopy data is versatile and provides a platform for systematic analysis of starvation responses in intact yeast cells.

## Results

### A convolutional neural network to classify vacuolar phenotypes from fluorescence images

**Figure 1.**
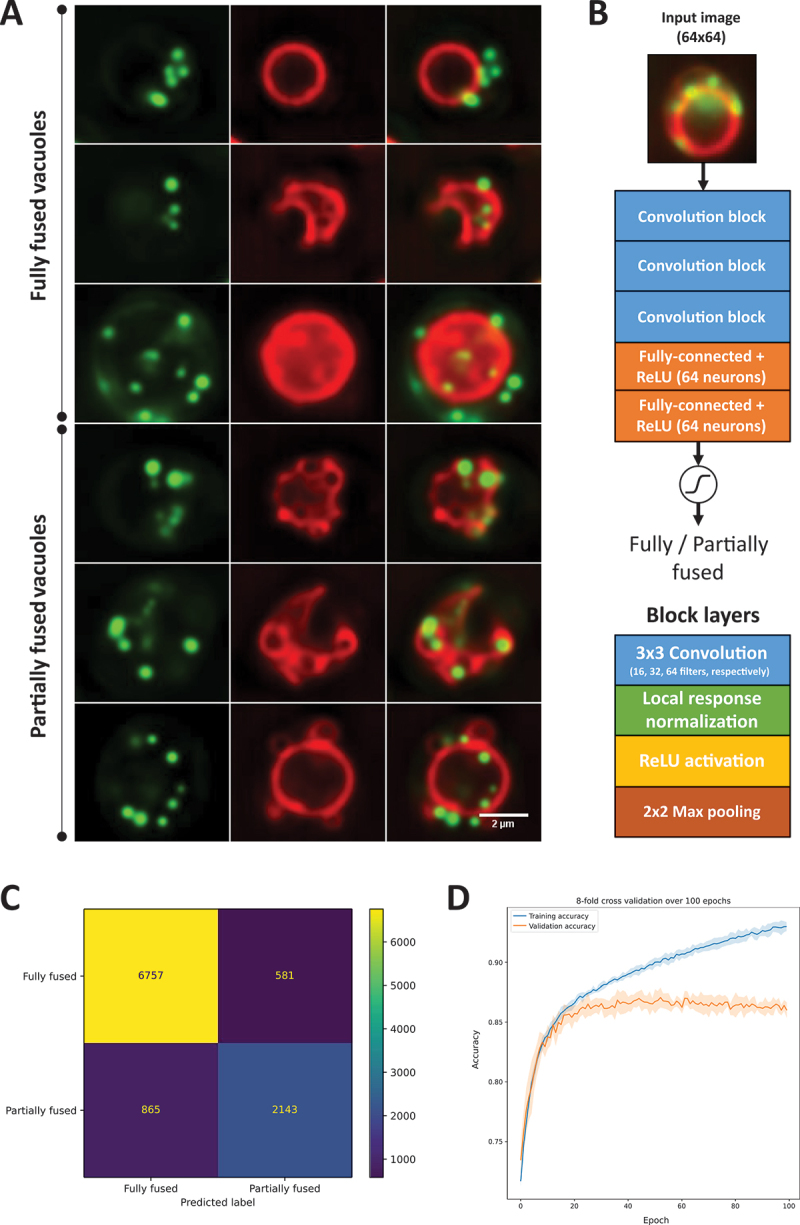
Deep learning-based classification of vacuole phenotypes from multicolor fluorescence images. Wild-type, *ncr1∆* and *npc2∆* cells were labeled with the ergosterol analog DHE (not shown) under anaerobic conditions in the presence of the vacuole marker FM 4–64 overnight, washed and labeled with BODIPY493/503 to stain LDs for 13 min, washed and imaged. (A) example montages of fully fused vacuoles (upper three rows) and partially fused vacuoles (lower three rows), defining the two phenotypes, which were annotated and used to train the DYFNet CNN model. Images of each channel (BODIPY493/503 in green; left column and FM 4–64 in red; middle column) were deconvolved before merging for visualization purposes only, but not for model training. The right column shows a color overlay of both channels. (B) architecture of DYFNet using 64 × 64images as input. Three convolutional blocks (each containing 3 × 3convolution, ReLU, local response normalization and max pooling) are followed by 2 fully-connected layers with 64 neurons and ReLU activations. A sigmoid activated neuron is used for the final binary decision of “fully fused” versus “partially fused” phenotypes. (C) confusion matrix of model output. The following metrics are derived from the confusion matrix: Accuracy = 86.02%, sensitivity = 71.24%, specificity = 92.08%, PPV = 78.67%, NPV = 88.65%, MCC = 65.29%. (D) accuracy for training (blue) and validation data (orange) as function of epochs upon 8-fold cross validation. See main text for further details.

When yeast cells are kept in the same culture medium during stationary growth, they become depleted of nutrients, resulting in several starvation responses, including vacuole fusion and lipophagy, the ingestion of LDs rich in sterol and triacylglycerol esters into the vacuole [2,10]. We have shown previously using fluorescence microscopy, that Ncr1 and Npc2 are instrumental for transport of sterols into the vacuole during stationary growth [8]. Absence of functional Ncr1 and Npc2 proteins diminished incorporation of the fluorescent ergosterol analog dehydroergosterol (DHE) into the vacuolar membrane, which was labeled with the lipophilic dye FM 4–64. While DHE initially traffics from the PM to LDs stained with BODIPY 493/503 in wild-type cells as well as in Ncr1- and Npc2-deficient cells, it is properly inserted into the vacuole membrane only in wild-type cells [8]. In *ncr1∆* cells and *npc2∆* cells DHE accumulates instead in LDs, and the vacuole often appeared lipid-filled with diffuse intravacuolar staining with FM 4–64 and intralumenal vesicles [8]. The latter could be autophagic vesicles inside of vacuoles, since it has been shown that FM 4–64 can slowly diffuse into the vacuole and label intralumenal membrane structures, including autophagic bodies [35]. Some FM 4–64 could also be invaginated during microautophagy, as has been shown in intact cells and with purified vacuoles [36,37]. Vacuoles of starved cells lacking either Ncr1 or Npc2 were often found to consist of several adherent vesicles instead of one central vacuole, as was mostly found in wild-type cells [8]. Vacuoles consisting of multiple clustered vesicles are normally found during exponential growth but indicate defective or delayed vesicle fusion, when they are found in starved cells, since during starvation vacuole vesicles normally fuse in yeast into one central vacuole [9,10]. To determine, whether absence of either Ncr1 or Npc2 leads to defects in vacuole fusion, we trained our DYFNet classification CNN which should distinguish two phenotypes: a “fully fused” state with one central vacuole (Figure 1A, upper three panels), and a “partially fused” state characterized by multi-vesicular vacuoles with either several small vesicles attached to each other, or one central vacuole with multiple attached vesicles (Figure 1A, lower three panels). The fully fused state could resemble either a circular or differently shaped vacuole (Figure 1A upper two panels).

**Figure 2.**
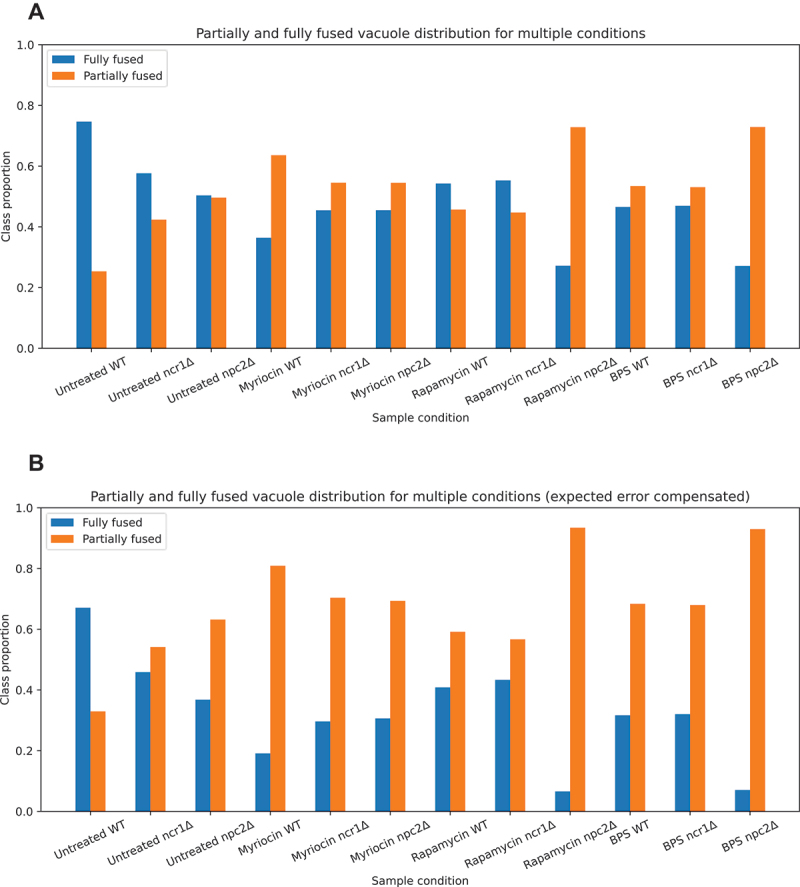
Model prediction for vacuole fusion under various conditions. (A) classification of fully and partially fused vacuoles in wild-type, Ncr1- and Npc2-deficient yeast cells. Wild-type, *ncr1∆* cells and *npc2∆* cells were left untreated or treated with myriocin, rapamycin or BPS as described in Materials and methods. Fluorescence images of cells in the green (BODIPY493/503) and red channel (FM 4–64) were acquired automatically and merged before classification of vacuoles. The results of the DYFNet classification is shown from left to right with fully fused vacuoles in blue and partially fused vacuoles in orange for untreated wild-type cells (*n* = 16288), *ncr1∆* cells (*n* = 5550) and *npc2∆* cells (*n* = 14248), for myriocin treated wild-type cells (*n* = 14251), *ncr1∆* cells (*n* = 8517) and *npc2∆* cells (*n* = 8734), for rapamycin treated wild-type cells (*n* = 2236), *ncr1∆* cells (*n* = 10270) and *npc2∆* cells (*n* = 9308) and for BPS treated wild-type cells (*n* = 3003), *ncr1∆* cells (*n* = 3501) and *npc2∆* cells (*n* = 3101). (B) an error compensated chart corrected based on the sensitivity and specificity of the rejection classifier and fusion classifier. The sensitivity and specificity are taken from the confusion matrix from 8-fold cross-validation and assumes equal error-rate across all sample conditions. All pairwise differences were found to be significant with *P* < 0.5%. P-values were empirically calculated by assessing the probability of randomly observing the given difference of means across distributions sampled 100.000 times from the confusion matrix, using a population size equal to the smaller of the two compared populations.

Vacuole fusion and lipophagy do not rely on the same molecular machinery [2,11,38,39]. Still, both processes are related via lipid transport activities, as for example Fat1, a fatty acyl coenzyme A synthetase and fatty acid transporter, was recently shown to regulate vacuole fusion and morphology but also lipophagy in stationary phase [40]. When assessing the relationship between droplet uptake and vacuole morphology, we found that fluorescence of the droplet marker BODIPY493/503 is inside and outside of the vacuole for both, fully and partially fused vacuoles (Figure 1A). Presence or absence of LDs in a vacuole was therefore not included in the phenotype annotation, though the green channel image of BODIPY stained LDs was included in model training. A portion of cells could not be annotated into those two categories, for which a third category, named “rejected”, was generated. Training and validation data was manually annotated into three categories: 1) cells with fully fused vacuoles (*n* = 7338), 2) cells with partially fused vacuoles (*n* = 3008) and 3) rejected cells (*n* = 8016). Training data was generated by locating regions of interest in the image fields based on identified peaks in the circular Hough transformed image. The regions are 100 × 100and 50 × 50for our Nikon and Leica acquisitions, respectively, and are later rescaled to 64 × 64using bicubic interpolation. This region of interest (ROI) extraction process also yields many ROIs without cells and uncentered, damaged, or poorly labeled cells, which are then annotated as “rejected”. Also, since the majority of image data was generated in an automatic fashion, a significant number of cells was out of focus, which also contributed to the rejected category. The DYFNet model architecture is based on the architecture proposed by Zhang et al. [12]. It contains 3 blocks of convolution, ReLU activation, local response normalization and max pooling followed by two fully-connected ReLU activated layers and finally a single sigmoid activated neuron (Figure 1B). The convolutional layers have a kernel size of 3 × 3and 16, 32, 64 filters respectively. Local response normalization, originally introduced in the ImageNet classification architecture, AlexNet [41], was added as a regularization layer, and has been shown to be useful in cell classification [12]. The fully-connected layers contain 64 neurons and the sigmoid function is used to assign a probability of belonging to the “partially fused” class. The model was compiled with the Nadam optimizer and binary cross-entropy loss [42]. A confusion matrix for assessment of model performance is shown in Figure 1C. This yields a sensitivity of 71.24% and specificity of 92.08% meaning that the model is considerably better at identifying the fully fused compared to the partially fused phenotype. The mean validation accuracy over 8-fold cross-validation converged to ~ 86% and is shown in Figure 1D. The arguably subpar accuracy is likely the result of forcing a complex phenotype into a binary annotation. This is supported by classification of the 64 features extracted in the last fully-connected layer for each phenotype using principal component analysis/PCA and stochastic neighbor embedding/t-SNE. Given the ambiguity of many samples, we have a reasonable separation of the phenotype-specific features (Figure S1). The model was used to predict the phenotypes of wild-type, *ncr1∆* and *npc2∆* cells from images, not included in the training data (>6000 per condition). This revealed a large percentage of fully fused vacuoles in wild-type yeast (i.e., 74.66%; Figure 2A). In *ncr1∆* and *npc2∆* cells, only 57.64% and 50.37% of vacuoles were fully fused with the remaining being partially fused. When correcting our model prediction for the miss-classification error obtained from the confusion matrix and assuming a constant error rate over the individual conditions, we find that severity of the vacuole fusion defect in *ncr1∆* and *npc2∆* cells is even larger (Figure 2B). All differences between conditions in both, the uncorrected and corrected model predictions are significant with *P* < 0.5%. These results show that yeast lacking functional Ncr1 or Npc2 protein have a compromised ability for vacuolar fusion. This could be the direct consequence of impaired delivery of ergosterol into the vacuole membrane in the absence of the Ncr1-Npc2 transport system, in line with our previous results, and the absolute requirement of ergosterol for vacuole fusion [8,43,44]. Alternatively, it could be the consequence of other factors, such as a disturbed sphingolipid homeostasis, altered TORC signaling or changed iron availability, which all have been observed in *ncr1Δ* cells [16,17,45,46]. To explore such possibilities, we have used inhibitors of the aforementioned processes and assessed their impact on vacuole fusion status in wild-type cells and in cells lacking either Ncr1 or Npc2 under starvation conditions. We found that inhibiting sphingolipid synthesis with myriocin, an inhibitor of serine palmitoyl transferase catalyzing the first step of sphingolipid synthesis, strongly increased the percentage of partially fused vacuoles. This effect was most pronounced in wild-type cells, where the contribution of multi-vesicular vacuoles went from 25.34% in the absence to more than 60% for the uncorrected data (Figure 2A) and 80% for the corrected data (Figure 2B) in the presence of myriocin. This result confirms earlier observations, which showed that sphingolipids and very long chain bases are essential for vacuole fusion [17]. Interestingly, myriocin had a slightly milder effect in both *ncr1Δ* and *npc2∆* cells (Figure 2). This could indicate that sterol transport by Ncr1 and Npc2 acts in concert with transport of sphingolipids, and both lipids together are essential during vacuole fusion.

Acute inhibition of target of rapamycin complex 1 (TORC1), a serine/threonine kinase controlling transcriptional programs for adaptation to changing nutrient conditions, has been shown to induce starvation responses in yeast but also to inhibit cell division [47]. Chronic exposure to low-dose rapamycin leads to expansion of chronological and replicative lifespan and to alterations in amino acid metabolism in yeast [48,49]. Mammalian cells lacking functional NPC1 have defects in autophagy and mitochondrial activity, which are linked to hyperactive TORC1 signaling as a consequence of cholesterol accumulation in the lysosomes [50]. Inhibition of TORC1 signaling in those cells, for example by rapamycin, restores lysosomal and mitochondrial function [51]. Conversely, knockdown of NPC1 or NPC2 in endothelial cells has been shown to cause decreased TORC1 signaling, and TORC1 activity was found to be reduced in yeast cells lacking Ncr1 [46,52]. Based on these findings, we assessed the effect of chronic inhibition of TORC1 activity on vacuole morphology in starved yeast lacking functional Ncr1 or Npc2. To this end, wild-type, *ncr1∆* and *npc2∆* cells were exposed to low doses of rapamycin for 48 h, and the extent of vacuole fusion was determined with our DYFNet CNN model. We found that rapamycin increased the percentage of partially fused vacuoles in all three cell types, but the effect was strongest in cells lacking Npc2 and smallest in Ncr1-deficient cells (Figure 2A,B). These results point to important differences in TORC1 signaling in cells lacking either Ncr1 or Npc2, which should be explored further in future studies.

Alterations of iron homeostasis have been observed in mouse models of NPC disease with increased iron content in the brain, which likely is part of the observed increase in oxidative stress in this disease [53,54]., Yeast cells lacking functional Ncr1 have increased iron content in their vacuole during stationary growth [46]. While iron depletion using specific brain-penetrable chelators does not improve neuronal function in NPC1-deficient mice, iron chelation using bathophenanthrolinedisulfonic acid disodium salt hydrate (BPS) increases the lifespan and restores autophagy in Ncr1-deficient yeast cells [46,53]. When treating cells with the iron chelator BPS, we found a decrease in fully fused vacuoles under all conditions (Figure 2). Thus, although iron depletion can restore oxidative stress resistance and autophagic flux in *ncr1∆* cells [46], it does not rescue the vacuole fusion defect but rather exacerbates it in cells lacking functional Ncr1. Interestingly, the increase in partially fused vacuoles upon BPS treatment was even more pronounced in cells lacking Npc2. Iron deficiency not only changes the pattern of sterols in yeast membranes but also results in reduced synthesis of sphingolipids and triacylglycerols and altered composition of LDs in yeast [55]. Our results indicate that one or several of these factors is/are essential for vacuole fusion in the absence of Npc2, such that cells depleted of iron have an even more severe phenotype. Future studies are needed to identify the molecular basis for this observation.

### Deep learning-based segmentation of LDs and vacuoles using fluorescence and X-ray image data

Fully and partially fused vacuoles sometimes appeared lipid-filled as judged by intravacuolar FM 4–64 staining, either as vesicles or diffuse labeling over the entire vacuole lumen (see Figure 1A, third row from top for an example). To identify lipid-filled vacuoles unequivocally, FM 4–64 staining detected by wide field fluorescence microscopy is not sufficient, and much higher resolution is needed. This is particularly true in cases of small vacuoles, in which out-of-focus fluorescence from the vacuolar membrane can obscure the intensity distribution of FM 4–64 in the image plane. We previously employed confocal microscopy to overcome this limitation, as it has better resolution particularly along the optical axis [8]. Still, confocal imaging is diffraction-limited, and staining with FM 4–64 can only report about the distribution of labeled membranes without providing a structural context. Here, we find that X-ray microscopy is ideal for this task, as it provides information about the cellular ultrastructure with an isotropic resolution of about 30 nm throughout the entire cellular volume for many cells in a reasonable amount of time. X-ray contrast is high for lipid membranes and intravacuolar lipid dispositions, allowing for a detailed analysis of lipophagy and other autophagic processes. To acquire the 3D tomograms, the sample is placed on a special holder that is tilted around a rotation axis. The raw tilt series is subsequently aligned, and 3D images of cells are reconstructed from the aligned tomograms. Yeast cells can be additionally labeled using suitable fluorescent organelle markers, and fluorescence images of such markers can be acquired in parallel to the X-ray tomograms on the same setup [32,56,57]. We employ this strategy to identify LDs using the droplet marker BODIPY 493/503 (emitting in green) and the vacuole marker FM 4–64 (emitting in red). A typical workflow for preparing and imaging yeast cells by combined fluorescence and SXT is shown in Figure S2.

**Figure 3.**
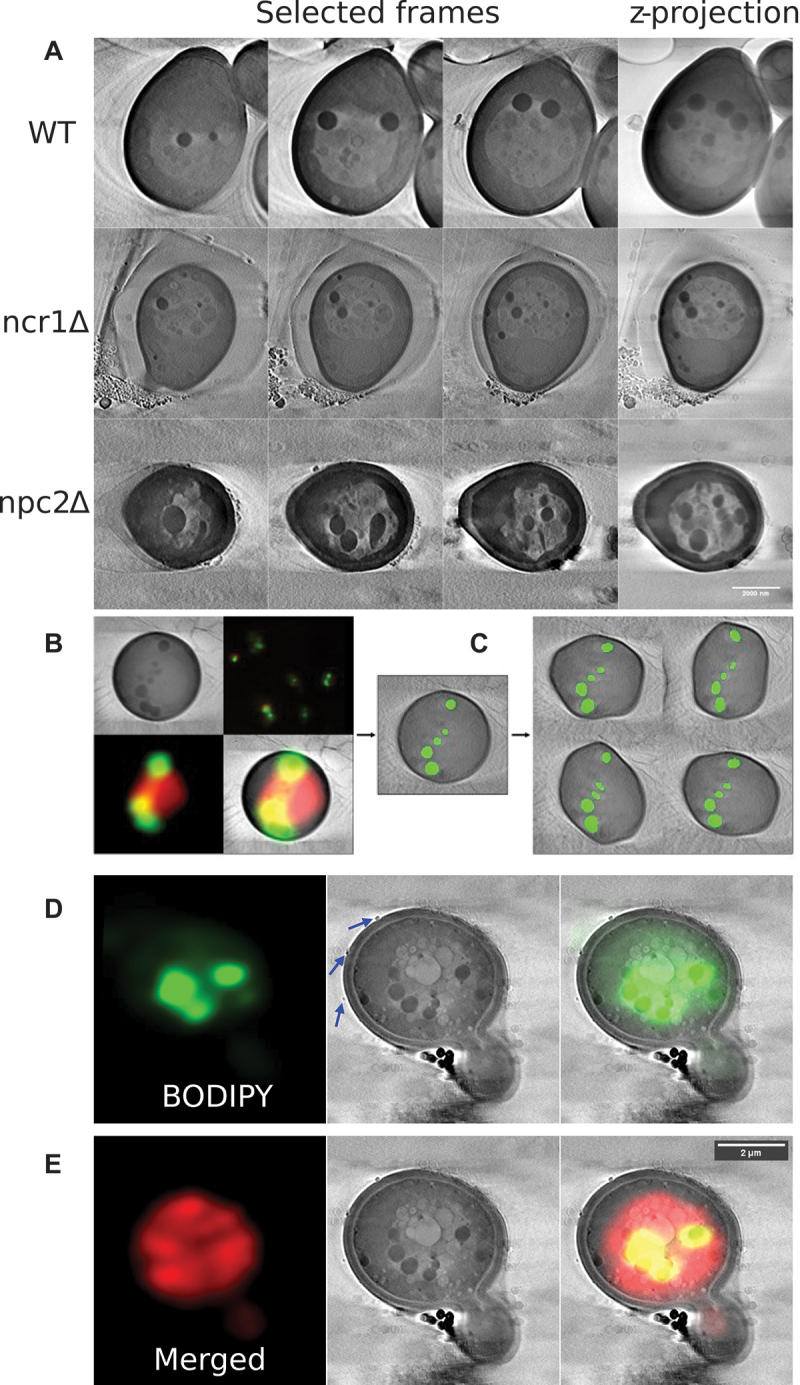
Correlative X-ray and fluorescence microscopy of wild-type, Ncr1- and Npc2-deficient yeast cells. (A) selected frames (left) and z-projections (right) of SXT reconstructions for wild-type (upper row), *ncr1∆* (middle row) and *npc2∆* cells (lower row) are shown. (B) correlative fluorescence and X-ray microscopy exemplified for a wild-type cell; upper left shows X-ray image, upper right, fluorescence image in original size, lower left is zoomed fluorescence image with BODIPY 493/503 in green and FM 4–64 in red and lower right is a color overlay. LDs are identified by the large X-ray absorption and in parallel by their green fluorescence from the BODIPY 493/503 staining. (C). Assuming presence of green staining, lipid droplets are annotated by hand and eventually augmented during training, to increase generalization and ground truth data efficiency. (D and E) correlative fluorescence and X-ray microscopy of *npc2∆* cells with a fragmented, i.e., multivesicular vacuole. Co-staining of X-ray image (middle panels) with BODIPY493/503 (D) and additionally with FM 4–64 (E) is shown. Right panels are overlays of fluorescence and X-ray images. Blue arrows in the middle panel of (D) point to structures resembling extracellular vesicles.

Wild-type yeast have typically one large vacuole under starvation conditions to which LDs can dock before being ingested (Figure 3A, upper panel). In addition, we find intralumenal vesicles and small droplet-like structures inside the vacuole of wild-type cells, but mostly in relatively low abundance. In cells lacking functional NCR1 more lipid material is deposited inside vacuoles, while in *npc2Δ* yeast cells, lacking functional NPC2 protein, the vacuole appears additionally deformed in many cells, as seen by SXT (Figure 3A, middle and lower panels). LDs appear as round and particularly dark spheres in SXT, as they efficiently absorb X-ray radiation, due to their high carbon and low water content, giving them a high linear absorption coefficient [28,30]. Differences in X-ray absorption and morphological characteristics are necessary conditions for organelle annotation, but they are often not sufficient criteria to unequivocally annotate subcellular structures in X-ray microscopy. For that reason, we co-labeled cells with FM 4–64 and BODIPY493/503, allowing for unequivocal identification of vacuoles and LDs in both imaging modalities (Figure 3B). To enrich the training data for the segmentation CNN model (see below), the input frames are augmented using random rotations and elastic deformations as described by Simard et al. (2003) [58]. This method generates variations of the original X-ray images, thereby greatly reducing the burden of manual image annotation for generation of training data (Figure 3C and below). In some of the Ncr1- and Npc2-deficient cells, aberrant vacuole morphologies were also seen by SXT. This is illustrated for *npc2Δ* yeast cells, in which vacuole staining by FM 4–64 is found in convoluted structures, which are clearly visible as multiple vacuole vesicles at the ultrastructural level by SXT (Figure 3D). LDs are found inside and attached to those fragmented vacuoles in *npc2Δ* cells while intense staining of LDs with BODIPY493/503 is found in the same region. Similar results were found for some *ncr1∆* cells (not shown). The majority of fully fused vacuoles in *ncr1∆* and *npc2∆* contained extensive intralumenal membrane accumulation. The intravacuolar lipid enrichment causes the extensive staining of FM 4–64 over the entire vacuole lumen in wide field fluorescence images, which we, however, did not include as criterion in the DYFNet classification model. This is, because vacuoles partially out of focus could also appear as lipid-filled in our automated fluorescence imaging setup. Here, X-ray microscopy really provides complementary information, as it allows for determining intravacuolar lipid assemblies in an unbiased manner. Together, these results show the potential of combined SXT and fluorescence microscopy to study the phenotype of yeast vacuoles and lipid storage in control, Ncr1- and Npc2-deficient cells.

**Figure 4.**
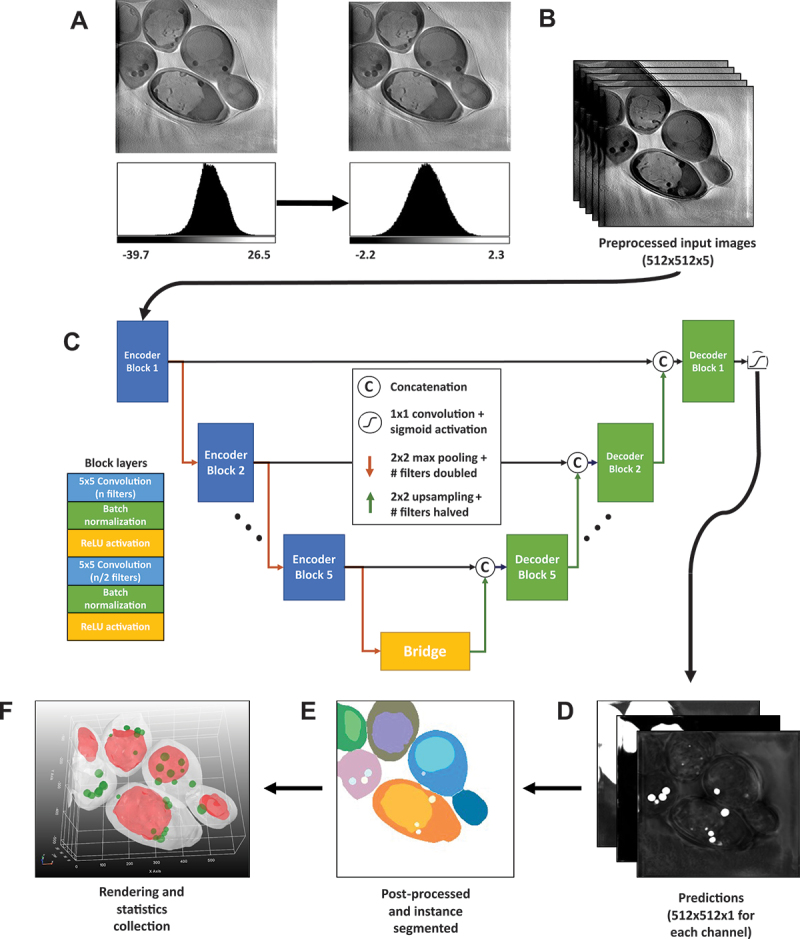
Workflow of LipoSeg for automated segmentation and architecture of the segmentation CNN. (A) to obtain comparable intensity statistics for all image data sets, tomogram reconstructions were first normalized using quantile normalization based on a gaussian distribution. (B) for model training, five sequential input slices were used for each stack of the training data and augmented by 90-degree rotations and elastic deformations. (C) the segmentation CNN model is based on the U-net architecture but with five sequential frames from a z-stack, which we found to minimize problems caused by the missing wedge problem. Each block consists of two iterations of convolution layers, batch normalization and ReLu activation unit, and blocks are connected by 2 × 2max pooling layers during the encoding phase (red arrows), and 2 × 2up-sampling layers during the decoding phase (green arrows). A sigmoid activation function is used as output, and each channel, i.e., entire cells, vacuoles and LDs, is trained as a separate model. During training, a compound loss function consisting of Dice and TopK binary cross-entropy is employed. (D) raw predictions for each channel are generated with probability of belonging to a given class, e.g., LDs, shown in grey scale intensity values. This is followed by instance segmentation using a watershed transformation for all three channels separately (E). The segmented stacks are then used for 3D rendering using a marching cube algorithm for mesh generation (F). This is shown here as top view of model output after 3D rendering showing cells in white, vacuoles in red and LDs in green exemplified for *npc2∆* cells. Finally, the segmented 3D objects (LDs and vacuoles) are analyzed including calculation of volumes, distances etc. See main text for further details.

To characterize the NPC-phenotypes at the ultrastructural level further and potentially discover further differences to control cells, the LipoSeg pipeline was created for instance segmentation, rendering and analysis of the X-ray images (Figure 4). The first step of the pipeline is comprised of a quantile normalization to achieve comparable image histograms of the reconstructed X-ray image stacks (Figure 4A). The input for the model consists of five sequential frames from a z-stack to aid in consistency across each tomogram reconstruction, thereby alleviating issues caused by the missing wedge problem (Figure 4B). The architecture of LipoSeg’s segmentation CNN is largely based on the original U-Net with a few changes (Figure 4C) [59,60]. The output is a single convolutional filter with a sigmoid activation; hence, each channel is trained separately with its own set of weights. This results in a modular pipeline and simplifies the training process as each channel can have its own subset of training data and model architecture. Additionally, trained channels can be used as pre-training for other channels, which can reduce the required training time. Models were trained for cell membrane, vacuole and droplet segmentation. During training, a compound loss function consisting of Dice and TopK binary cross-entropy is used, with the K parameter available as a user parameter in the pipeline. Each training/label pair is sampled equally with respect to the biological sample, to prevent overfitting. Output of this model are the predictions for each channel (Figure 4D).

To ensure robustness on a model trained on a smaller training dataset, optional post-processing steps were added. The options are outlier removal, masking, averaging/blurring, hole filling, convex constraints, minimum volume filtering and surface smoothing. Combinations of these options were used to generate new training data from unseen data, and many options are recommended, even on a well-trained model [60–62]. Given that the accuracy of the segmentation has been convincing, it discharged us from creating further manual segmentations. During development and training data creation, we tested an earlier version of the models using unseen, and partially seen test data, which has since been added to the training data. We calculated the Dice similarity coefficient (DSC) for each frame over two fully hand-segmented z-stacks (see Materials and Methods) [63]. We observe a DSC of > 0.9 in most frames for the cell membrane segmentations, with some exceptions in the first and last frames, where the missing wedge problem leaves the segmentation ambiguous (Figure S3A, B, black curves). The same pattern is observed in the droplet channel, in which the DSC oscillates for the same reason (Figure S3A, SB, green curves). In most cases, the prediction of the model fits better visually, than the hand-annotated training data, explaining many of the misclassifications (Figure S3C and SD). The good agreement between X-ray images and model prediction can also be seen in the Video S1.

Instance segmentation is conducted using a distance transform watershed based on the implementation in MorphoLibJ, which implements a workflow using Meyer and Beucher’s watershed algorithm [64,65]. Watershed was favored over a machine learning approach using bounding boxes or separate neural networks, such as Mask R-CNN used by Li et al. (2022) [34], to minimize constraints and workload on training data. We found that our approach was sufficient for yeast cell images, where the visible cellular morphology generally resembles ellipsoids.

A mesh is generated from the instance segmentation using marching cubes and optionally smoothened (Figure 4E,F). The meshes are rendered in an interactive 3D environment using PyVista [66]. Each mesh is saved for future rendering or analysis. Analysis could include measurement of volume, distance to nearest droplet, vacuole or cell membrane and containment of other meshes (i.e., whether LDs have been consumed by a vacuole). A representative 3D rendered image of Npc2-deficient yeast cells is shown in Figure 4F. Our model allows for accurate segmentation and 3D rendering of vacuoles and LDs in the majority of cells in a fully automated manner. Note that fragmented vacuoles or vacuoles in cells located at the edge of the tomograms could not be identified by our method. As a result, in some cells, LDs but no vacuole could be detected, while occasionally some cells had no visible droplet structure (see examples in left two cells of Figure 4F). Such cells at the edge of the images were discarded from further analysis. In most cells having fully fused vacuoles, however, both, vacuoles and LDs were identified and 3D-rendered and appear well-separated from each other in the 3D representation (Figure 4F, central two and right two cells).

**Figure 5.**
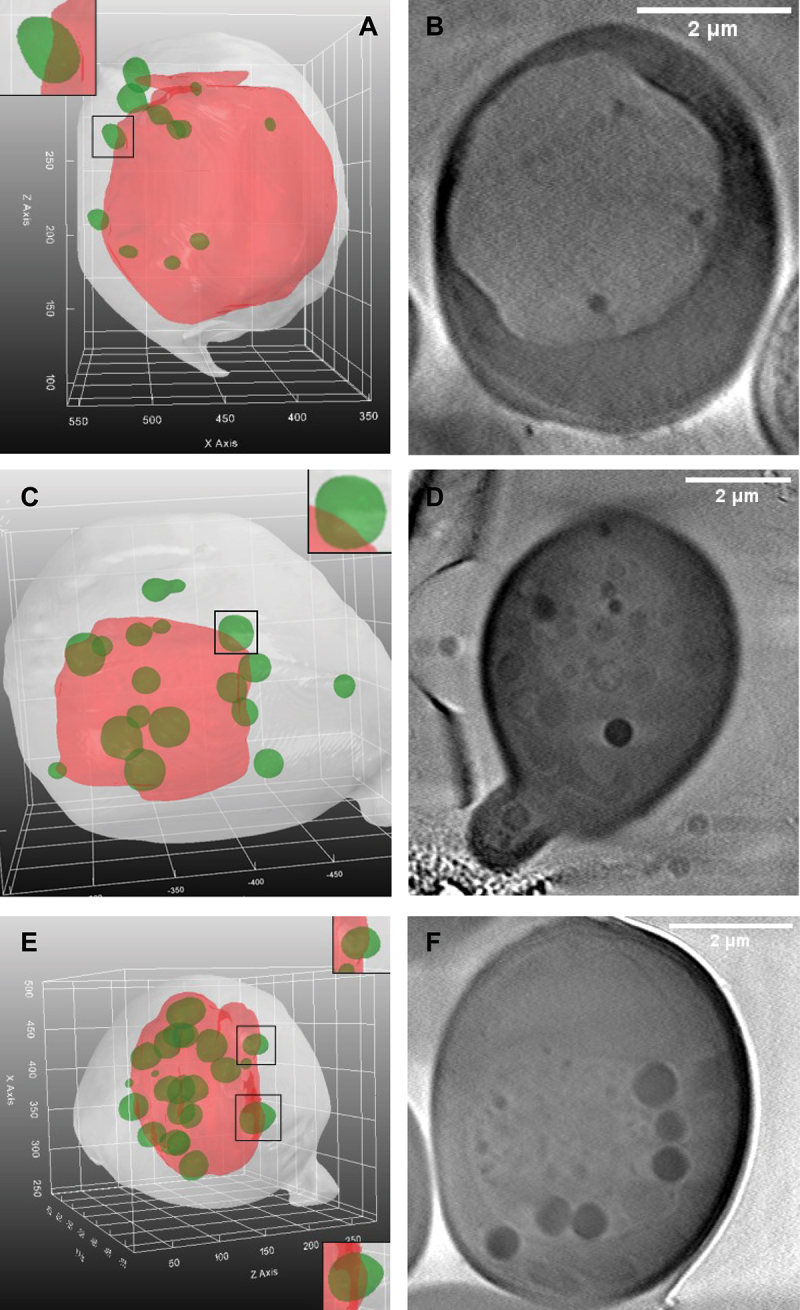
Vacuolar shapes from 3D renderings for wild-type cells and for yeast lacking either Ncr1 or Npc2. 3D renderings as obtained from the segmentation CNN model for wild-type cells (A), *ncr1∆* cells (C) and *npc2∆* cells (E). Insets show zoomed versions of rectangular black boxes highlighting green LDs docking on the surface of the vacuole, shown in red. Corresponding10 slice sum projections for X-ray reconstructions from corresponding tomograms for wild-type cells (B, *n* = 20), *ncr1∆* cells (D, *n* = 11) and *npc2∆* cells (F, *n* = 5). See main text for further details.

Lipophagy starts by docking of LDs at distinct sites of the vacuole surface, and those domains are supposed to be highly ordered, rich in ergosterol and certain proteins [18,67]. Lack of Ncr1 or Npc2 was suggested to lower formation of such vacuolar domains, thereby diminishing lipophagy, which was attributed to defective ergosterol transport [7]. However, this could not be confirmed in another study using acute glucose restriction [68]. Both, blocking ergosterol synthesis and ergosterol overloading by preventing its esterification, seem to prevent formation of vacuolar domains in living cells, suggesting that a fine sterol balance is needed to maintain the correct vacuolaor ultrastructure during starvation [68,69]. Experiments on isolated vacuoles show that vacuolar domains only form upon ergosterol depletion, and they provide evidence that vacuoles only contain about 10 mol% of ergosterol in both, the logarithmic and stationary phase [70,71]. Based on these interesting but somehow contradicting observations, a coherent picture of vacuolar phenotypes of *ncr1∆* and *ncr2∆* yeast cells is lacking. Our model allows for 3D visualization and assessment of vacuolar structure and the spatial arrangement of LDs in wild-type and mutant cells, as shown in Figure 5A–F. For this, no manual segmentation or correction is needed, but 3D renderings can be obtained fully automatically with only X-ray tomogram reconstructions as input. Cells and their vacuoles appear sometimes cut off at the top or bottom as seen in side-views of 3D renderings. This is an artifact of the limited range of recorded tomographic projections, which is between ±80 degrees but due to the ice thickness of the cryo-frozen sample maximally from −65 to + 65 degrees at the BESSYII facility (see Materials and Methods). Ideally, one would like to aqcuire a full-range tomogram, which requires acquistion from −90 to + 90 degrees, however, this is impossible with the current sample holder and given hardware set up. The reduced number of projections compared to a full tomogram leads to the so-called missing wedge or missing cone, a well-known problem in computerized tomography, for which currently no satisfying algorithmic solution exists. The problem is illustrated in 2D for a simulated yeast phantom image for the filtered back projection algorithm used in this study (Figure S4). One finds that the reduced number of projection angles, or missing wedge, causes shading artifacts at the edge of the simulated LDs, vacuoles and cell borders. The severity of such artifacts depends on the orientation of the object in the simulated image relative to the projection axis and on the distance from the image center. As a consequence, these artifacts affect the segmentation quality to varying degrees, depending on the position and orientation of the yeast cells in the ice of the cryo-sample relative to the radiation beam. The diminishing contrast at the edge of the vacuole, for example, can result in segmentation errors and cutting of the object, as seen in some 3D reconstructions (see an example in Figure 5C,D). The same problem can also occasionally result in some mismatch between subjectively assessed manual segmentation of LDs and model output (Figure S3).

For wild-type cells, as well as for *ncr1∆* and *npc2∆* cells, we observed docking of LDs to vacuoles, resulting in slight indentation of the vacuole membrane at the docking site (Figure 5A,C,F). Such indentations have been observed previously by freeze-etch EM, and based on fluorescent markers in confocal microscopy, and those regions have been suggested to be enriched in certain proteins and lipids, necessary for droplet docking and ingestion [7,18,69]. The fact that we observed docking sites for LDs on vacuoles with similar frequency in wild-type cells as in *ncr1∆* and *npc2∆* cells argues against defects in the initial stage of lipophagy in cells lacking functional Ncr1 or Npc2. From the 3D renderings, one can also observe the pseudo-hexagonal structure of the vacuoles, which is characteristic for the micron-scale domains found in vacuoles of starved cells [7,18,69,72]. This can be clearly discerned in side-views of 3D rendering, especially for wild-type and Ncr1-deficient cells. In *npc2∆* cells we also find hexagonal-like vacuolar shapes but often additionally crinkles and an overall buckled structure (Figure 5E,F and Figure S5). In addition, vesicles and tubular membrane structures were found inside vacuoles, and those were particularly pronounced in *npc2Δ* cells (Figures 3A and 5F and Figure S5A-E) but also prominent in some *ncr1Δ* cells (Figures 3A, 5D and Figure S5F). In some *npc2Δ* cells, we observed LDs inside of intralumenal vesicles, as previously concluded from thin EM sections [18]. While the function of such vesicles is unknown, they might protect LDs from hydrolysis by intravacuolar hydrolases or they could be the result of a different autophagic mechanism, as previously suggested [18,73]. Most of these intravacuolar membrane assemblies are of low X-ray contrast, and this together with their heterogenous shape make them difficult to identify in an automated manner. They have therefore not been considered in our segmentation algorithm.

We also found, especially in *npc2Δ* cells, tiny vesicles at the cell wall, which are likely secreted from the cells (Figure 3D,E and Figure S5, blue arrows). Such extracellular vesicles (EVs) have been observed previously by EM, purified from cells, and analyzed by light scattering and cryo-EM tomography [74,75]. The EVs, we find occasionally in *npc2∆* cells by SXT are in the size range of 60–100 nm, which is in line with previous reports on EVs in yeast cells [76]. Such vesicles could be involved in cellular efflux of excess lipids, release of exosomes and autophagic membranes in yeast, as recently proposed [77]. Interestingly, using combined fluorescence and X-ray microscopy, we recently observed and characterized shedding of EVs from the plasma membrane of fibroblasts from NPC2 patients, and we showed that such vesicles contain abundant cholesterol and lysosomal cargo [56]. Thus, for both, yeast and mammalian cells, the high resolution provided by SXT allows one to study secretion of EVs. However, the rare occurrence of EVs in X-ray images of yeast makes a quantitative analysis difficult. Together, we conclude from this data, that yeast cells lacking functional Npc2 have a more severe phenotype than Ncr1-deficient cells, which is in line with the results of our classification CNN (see Figure 2).

**Figure 6.**
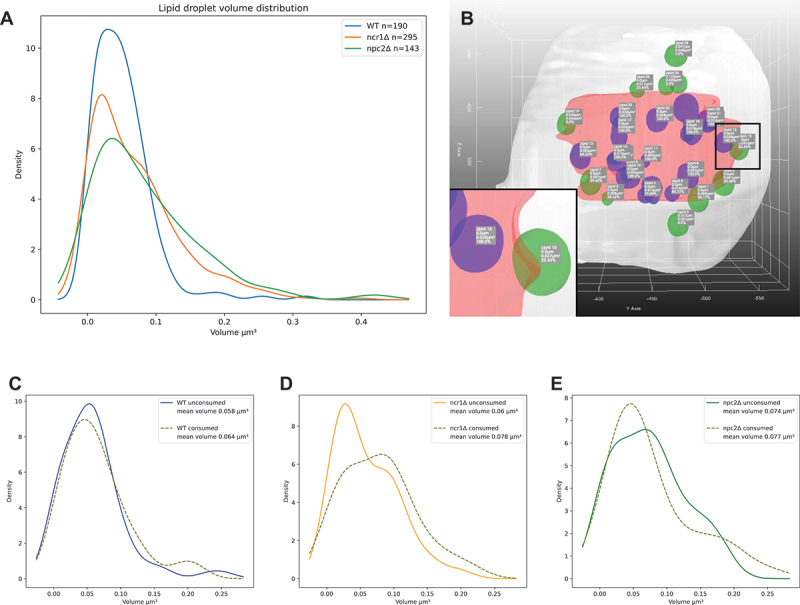
Quantitative analysis of droplet size and lipophagy in yeast cells from the LipoSeg pipeline output. (A) kernel density plot of droplet volume measured from 3D-rendered LDs inside and outside of vacuoles in wild-type cells (blue line), *ncr1Δ* cells (orange line) and *npc2Δ* cells (green line). Model output with LDs inside vacuoles color-coded in pink, and LDs outside of vacuoles in green (B). Labels are provided showing statistics for each droplet. The inset in (B) corresponding to the black box show one droplet inside of the vacuole in purple, and another one attaching in green. Identified LDs were separated into those outside vacuoles (“unconsumed”) and inside vacuoles (“consumed”), and their respective volume was calculated for wild-type cells (C), *ncr1∆* cells (D) and *npc2∆* cells (E) and plotted as kernel density plot. See main text for further details.

Using our segmentation pipeline, we are able to quantify the extent of lipophagy and droplet consumption from X-ray reconstructions. For that, the meshes of 72 yeast cells across three conditions were generated and analyzed. 3D renderings of wild type, *ncr1∆* and *npc2∆* cells (*n* = 24, 30, 18 cells respectively) were used to measure the volume and pairwise distances between LDs and vacuoles. Together, we found 686 droplet meshes (i.e., 245 LDs in wild-type cells, 298 in *ncr1∆* cells and143 in *npc2∆* cells, respectively; Figure 5A). Together, 28 central vacuoles could be segmented, and in those cells (*n* = 14, 16, 8, respectively), we found 272 LDs. Both, *ncr1∆*, and *npc2∆* cells had significantly larger LDs compared to the wild type as seen in Figure 6A. A Mann-Whitney U-Tests showed the significance of these results by giving p-values of 0.0104 and 0.0009 for *ncr1∆*, and *npc2∆* cells, respectively. Furthermore, our method allows for studying ingested LDs and LDs outside of vacuoles separately. LDs inside vacuoles are color-coded in purple in Figure 6B together with the key statistics for each droplet object. This includes a number for each droplet, the distance to the vacuolar membrane in µm, the droplet volume in µm^3^ and the percentage of its area in contact with the vacuole. Accordingly, LDs consumed by the vacuole have 100% of their surface area in contact with the vacuole, while those attaching on the outside have variable percentages, i.e., 22.43% for the green droplet in the inset of Figure 6B. LDs outside of the vacuole have of course 0% of their surface in contact and a given distance > 0 from the vacuole membrane. This analysis allows for determining contact formation between both organelles in 3D in a fully automated manner. Extensive droplet uptake into the vacuole was observed in all three cell types. When measuring droplet volumes for every condition in cells with intact vacuoles, we found that the droplet volume is slightly larger inside of vacuoles compared to LDs in the cytoplasm, particularly for *ncr1∆* cells (Figure 6C–E). However, the available data set is too small to allow us to conclude on the significance of this observation. It has been shown that lipid hydrolysis can take place inside but also outside of vacuoles in yeast, and that both processes can be coupled [78,79]. Thus, the significantly larger LDs, we observe in *ncr1∆* and *npc2∆* cells can either be due to impaired intravacuolar or cytosolic hydrolysis of sterol esters and triacylglycerols. Future studies are needed to clarify this matter. Together, our pipeline can identify differences in lipid mobilization from LDs between wild type, Ncr1- and Npc2-deficient yeast cells, which could not be identified without automated organelle segmentation of X-ray images and subsequent statistical analysis.

## Discussion

In this work, we developed a deep learning approach to automatize the analysis of fluorescence and X-ray microscopy images of yeast cells during starvation. We focused on two aspects of starvation responses, which both relate to the yeast vacuole: homotypic vacuole fusion and lipophagy. Fusion of vesicles into one central vacuole is an important process during starvation in yeast, enabling for efficient delivery of autophagosomes and other vesicles as well as for adaptation of the organelle volume to the increasing lipid content [9,10,80]. Lipophagy is a form of autophagy in which LDs are ingested into the vacuole during starvation. It is increasingly recognized as important regulatory process for maintenance of cellular energy homeostasis. Dysfunction of lipophagy has been shown to play an important role in development of obesity, development of type 2 diabetes and oxidative stress as well as in protein aggregation during neurodegeneration [81,82]. Lipophagy is often studied in yeast, *Saccharomyces cerevisiae*, because this model organism is straightforward to manipulate genetically and also because the yeast vacuole is large enough to be amenable to light microscopic investigation. This allows for counting the number of LDs inside vacuoles as a measure for lipophagy under various starvation conditions and genetic backgrounds [7,18,69,83,84]. Similarly, vacuole morphology is often assessed by visual inspection, and from the number of attached vesicles the fusion status is inferred [11,15,17]. While reliable and largely reproducible between different human observers, visual annotation of vacuole phenotypes is tedious. Given the recent progress in machine learning, in particular of deep learning in microscopy [85], we chose to implement a neural network for automatizing this task. In contrast to classical image analysis, CNNs can automatically optimize feature learning and extraction for phenotype classification by learning from annotated examples (so-called supervised learning). This approach has been used extensively for phenotype classification in yeast, including analysis of subcellular protein localization, cell morphology, cell budding, genetic interactions and autophagy based on light microscopy image data [12,86–88].

We show that cells with a single central vacuole stained with FM 4–64 can be automatically distinguished from cells with multiple vacuole vesicles by DYFNet, our classification CNN (Figure 1). This enables us to show that yeast lacking functional Ncr1 or Npc2 have a reduced capacity for vacuole fusion and/or increased vacuole fragmentation (Figure 2). This could be a consequence of defective ergosterol transport into the vacuolar membrane, as ergosterol is needed for vacuole fusion [8,43]. The complex vacuole phenotype, we observed in starved cells, limits the performance of the DYFNet model, which has a limited capability to separate the two phenotypes into clearly defined classes (Figure S1). Apart from fully versus partially fused vacuoles, we also found accumulation of lipids in the vacuole to a varying degree, which resulted in partial staining of FM 4–64 in the vacuole lumen. Such intralumenal lipid accumulation, however, is in many cases difficult to distinguish from vesicles attached to the vacuole, also for the human beings, who annotated the image data. Also, the partially fused state could be the result of several docked vesicles or incomplete fusion of smaller vesicles with a central vacuole, which we do not distinguish in the current model implementation. Finally, the vacuolar staining we use by labeling cells with FM 4–64 can result in heterogenous fluorescence patterns due to the formation of vacuolar domains during starvation, into which FM 4–64 partitions differently [18]. For these reasons, the vacuolar appearance can become multifaceted, which is strongly simplified in our binary classification.

Since diffraction-limited fluorescence microscopy lacks the resolution to study the vacuolar phenotype at the ultrastructural level, we employed a combination of fluorescence and X-ray microscopy for this task. We confirmed by X-ray microscopy that vacuoles of *ncr1∆*, and *npc2∆* cells sometimes appeared fragmented, showing that vacuole fusion is compromised in a subpopulation of cells lacking either Ncr1 or Npc2 (Figure 3D,F). We also found that *ncr1∆* and *npc2∆* cells deposit more intravacuolar lipid material than wild-type cells which is in line with the function of Ncr1 and Npc2 as lipid transporters out of the vacuole (Figure 3A) [8,13,14,89]. Vacuoles and LDs can be unequivocally identified by their characteristic shape and X-ray absorption but also by the overlap in fluorescence from organelle-specific markers, emphasizing the potential of correlative fluorescence and X-ray imaging (Figure 3B). We make use of another and more complex CNN to segment these organelles from X-ray reconstructions. CNNs have been used extensively for segmentation of computational tomography data in the clinical imaging communities [59–62]. However, few studies have used CNNs for segmentation of SXT data of individual cells [33,34,90]. Using a U-net based architecture for the segmentation task and a traditional, watershed-based approach for the subsequent instance segmentation, we keep the computational burden and need for training data at a minimum and are nevertheless able to automatically segment vacuoles and LDs from X-ray image stacks and thereby to quantify the extent of droplet uptake into the vacuole. Our LipoSeg pipeline thereby differs from previous attempts to segment organelles from X-ray tomogram reconstructions, as we use partially fluorescence annotated X-ray data for training [33,34,90]. Interestingly, we find that LipoSeg’s CNN model also allows for segmenting LDs in mammalian cells, as shown for a macrophage foam cell in Figure S6. Segmenting other organelles, like the lysosomes in mammalian cells would require their proper annotation in X-ray reconstructions for model training. This, however, is not possible without additional labeling using fluorescence markers since lysosomes cannot be so easily discerned as droplets or the yeast vacuole without co-staining. Also, segmenting entire mammalian cells from X-ray reconstructions is impossible due to the much larger size of these cells, which is larger than the field of view in the X-ray microscope. However, even without further training our model is well-suited to analyze droplet size and distribution in mammalian cells, which could find wide application for example to study changes in droplet size and abundance in atherosclerosis, lipodystrophies as well as neurodegenerative and liver diseases [82,91–94].

We find that *ncr1∆* and *npc2∆* cells can internalize LDs into the vacuole similar to control cells, however, the intralumenal LDs are enlarged in cells lacking functional Ncr1 and Npc2 (Figure 4). 3D rendering allows us not only to determine the extent of lipophagy but also to determine the surface fraction of the segmented LDs in contact with vacuoles. This is important, as it enables us to study contact sites between organelles in a fully automated manner from SXT data. SXT has previously been shown to allow for studying membrane contact sites between lysosomes and mitochondria and between insulin granules and mitochondria but only on a qualitative level or upon tedious manual segmentation [95,96]. Our quantitative analysis allows for determining the contact surface between LDs and vacuoles in 3D from X-ray images in an automated manner, which will be important for analysis of contact sites between other organelles in the future. The increased size of LDs in *ncr1∆* and *npc2∆* cells compared to wild-type cells raises the question, whether this is due to impaired intravacuolar droplet digestion or reduced lipolysis in the cytoplasm. While we cannot answer this question at the moment, we found that droplets inside the vacuole are slightly larger in *ncr1∆* and *npc2∆* cells (Figure 6C-E). Interestingly, cytosolic hydrolysis of stored triacylglycerols and ergosterol esters in the cytosol has been shown to be coupled to a functional vacuole, and the products of cytosolic digestion of LDs were found to be efficiently used for membrane remodeling during growth resumption [78]. It will be interesting to study, whether droplet utilization under growth resumption is differently affected by lack of Ncr1 or Npc2 compared to stationary phase lipophagy.

A recent study used freeze-etch EM to study lipophagy in cells lacking functional Ncr1 or Npc2 during nitrogen starvation and found evidence for impaired engulfment of LDs into the vacuole [7]. This was accompanied by reduced formation of vacuole domains in this study. In addition, autophagic vesicles inside the vacuole were found to be smaller in Ncr1- and Npc2-deficient yeast compared to wild-type cells [7]. We cannot confirm these findings, as droplet docking to the vacuole and engulfement of LDs does not seem to be the affected step in *ncr1∆* and *npc2∆* cells in our experiments. Normal droplet ingestion into vacuoles based on a biochemical assay (i.e., vacuolar degradation of Erg6 associated with LDs) was also found in another recent study [19]. Similarly, vacuolar domains, which are thought to be essential for droplet docking during lipophagy appear to be normal in *ncr1∆* and *npc2∆* cells during stationary phase and glucose starvation [19,68]. Since the mode of induction of lipophagy seems to affect the molecular machinery, which yeast cells use to execute lipophagy [2], it is possible that Ncr1 and Npc2 proteins are differently involved in stationary phase compared to other forms of lipophagy. In line with this hypothesis are recent findings, that lipid imbalance by disturbed PC synthesis and ER stress affect vacuole domain formation in an Ncr1- and Npc2-dependent manner during lipophagy [19].

While LDs are rich in ergosterol and yeast Ncr1 and Npc2 proteins are important for sterol transport into the vacuole membrane, Ncr1 and Npc2 might transport other lipids as well. In fact, we showed recently, that yeast Npc2 binds a variety of phospholipids and can adapt its binding pocket to the different lipid ligands [89]. If this is similarly the case for Ncr1, one could hypothesize, that both proteins are needed to shuttle a variety of lipids out of the vacuole during starvation. As a consequence, absence of either protein should slow intravacuolar lipid digestion, simply because hydrolysis products would not be exported from the vacuole and thereby could inhibit further lipid cleavage by product feedback inhibition. As a consequence, the altered lipid composition of the vacuolar membrane could result in reduced vacuole fusion resulting in the observed fusion defects in yeast lacking either Ncr1 or Npc2. We are currently testing this hypothesis using the tools described in this study. Apart from Ncr1 and Npc2, there are other proteins which are essential for both, vacuole fusion and morphology as well as turnover of LDs during stationary phase: Fat1 is well known to mediate import of fatty acids into yeast cells but it also locates to LDs and has recently been described to regulate vacuole fusion and morphology during starvation [40,97]. Since Fat1 also possesses very-long-chain acylation activity, it was suggested to control vacuole morphology, fusion and lipophagy by regulating the fatty acyl chain composition of phospholipids in the vacuole membrane [40,97]. Recent analysis of the lipid composition of vacuoles isolated from log- and stationary-phase grown yeast indeed found an increase in saturated phospholipid species during starvation [71]. Since mammalian NPC1 has also been implicated in fatty acid transport [98], it is tempting to speculate that the Ncr1-Npc2 system works in concert with Fat1 and other transporters to ensure a proper lipid composition and thereby function of the vacuole during starvation. Future studies are needed to unravel the molecular mechanisms underlying the function of Ncr1 and Npc2 in starvation responses in yeast, and the tools provided here will be instrumental for this endeavor.

## Materials and Methods

### Yeast strains, labeling and preparation for microscopy

The WT BY4741 yeast strain was obtained from the Euroscarf culture collection (Y00000), while the BY4742 *ncr1∆::KanMX4* and BY4742 *npc2∆::KanMX4* strains were kindly provided by Prof. Christopher Beh of Simon Fraser University, Burnaby, Canada. The cells were grown in YPD media consisting of 2% D(+)-glucose monohydrate (Merck, 1.08342.1000), 2% Bacto Peptone (BD Chemicals 211,677), 1% yeast extract (Merck, 1.03753.0500), and 0.02% adenine (Sigma-Aldrich, A-2786). Prior to experimentation, the cells were cultured in 5 mL of media in a 50 mL Falcon tube at 30°C with 150 rpm until they reached the stationary phase. The optical density at 600 nm (OD_600_) was then measured using a WPA Biowave CO8000 (Biochrom Ltd, UK), and the cells were washed three times with sterile water before being diluted to a final OD_600_ concentration of 0.1. The diluted cells were incubated for 22 h under anaerobic conditions in YPD media containing 0.1 µg/mL *N*-(3-triethylammoniumpropyl)-4-(6-[4-{diethylamino} phenyl] hexatrienyl) pyridinium dibromide (FM 4–64; Thermo Fisher, T3166) and 0.1% Tween 80 (Sigma-Aldrich, P4780) with 6 µg/mL DHE (Sigma-Aldrich, 810253P). After 22 h, the yeast cells were washed three times with PBS (phosphate-buffered saline; Thermo Fisher 70,013,016), which was a 10-fold stock composed of 15.44 mM KH_2_PO_4_, 1551.72 mM NaCl, 27.09 mM Na_2_HPO_4_-7 H_2_O, pH 7.2, being diluted with H_2_O to a final concentration of 1 × . After washing, cells were resuspended in the original growth media. The cells were treated with either myriocin (0.4 µg/ml in EtOH; Sigma-Aldrich, M1177), bathophenanthrolinedisulfonic acid disodium salt hydrate (BPS, 48 µg/ml in water; Sigma-Aldrich 146,627), rapamycin (1.8 ng/ml in DMSO; Sigma-Aldrich, R0395) or nothing and incubated in the aerobic conditions for 48 h. For imaging, the cells were diluted in PBS to a final OD_600_ concentration of 1.0 and labeled with 0.5 mg/mL 4,4-difluoro-1,3,5,7,8-pentamethyl-4-bora-3a,4a-diaza-*s*-Indacene (BODIPY 493/503; Thermo Fisher, D3922), a marker for LDs, first for 3 min in the tube before adding the solution to the microscope dish and allow it to settle for another 10 min.

### Culture and imaging of macrophages

J774 murine macrophages were purchased from ATCC (TIB-67) and cultured in DMEM culture medium (GIBCO BRL/Life Technologies, D6429) supplemented with 1% glutamine, 1% penicillin and 10% fetal bovine serum. For imaging, cells were seeded onto poly-lysine coated gold grids, as described below for yeast cells, washed with buffer medium containing 150 mM NaCl, 5 mM KCl, 1 mM CaCl_2_, 1 mM MgCl_2_, 5 mM glucose and 20 mM HEPES, pH 7.4 and treated for 6 h with acetylated low density lipoprotein (AcLDL), prepared as described previously [99]. After that, cells were labeled with BODIPY 493/503, as described above, washed with buffer medium and fixed with 2% paraformaldehyde before transfer to the HZB for imaging at the U41-PGM1 TXM beamline (see below).

### Fluorescence microscopy and soft X-ray tomography

All fluorescence images used for vacuole phenotyping by deep learning were acquired using two different wide-field microscopes. A Leica DMIRBE microscope with a 100 × 1.3 NA with an oil immersion objective and a Nikon Ti-2 Eclipse with a 100 × 1.4 NA with an oil immersion objective and post-magnification. Automated imaging was carried out on the Nikon microscope with a circular random selection of the positions within 2 mm using perfect focus. From each repetition (microscopy dish), 30 positions were found and imaged first using all selected channels (green, red and bright field) and then moving to the next positions until all 30 images were acquired. On Leica DMIRBE, images were obtained using a neutral density filter. For FM 4–64 dye a standard rhodamine filter set with a 535 nm (50-nm bandpass) excitation filter, 565 nm dichromatic mirror and 610 nm (75-nm bandpass) emission filter was used. BODIPY 493/503 was imaged using a standard fluorescein filter set, with a 470 nm (20-nm bandpass) excitation filter, 510 nm dichromatic mirror and a 537 nm (23-nm bandpass) emission filter. The fluorescent sterol, DHE, was imaged using a special design filter cube in the UV range with a 335 nm (20-nm bandpass) excitation filter, 365 nm dichromatic mirror and 405 nm (40-nm bandpass) emission filter on the Leica DMIRB microscope. On the Nikon Ti-2 standard FITC and TRITC filter cubes were used for imaging of BODIPY 493/503 and FM 4–64, respectively. For SXT, cells were treated as described above, seeded on the poly-D-lysine coated R 2/2 grids (Quanti-foil, 100 Holy Carbon Films, Grids: HZB-2 Au), and a small volume of 270 nm gold beads was added to the grids as fiducial markers. The sample was plunge-frozen and stored in liquid nitrogen before imaging. The imaging of the grids was done on the U41-PGM1 TXM beamline over a tilt angle range of 120–125° with 1° tilt steps using an X-ray photon energy of 510 eV and a 25-nm zone plate. Before SXT, fluorescence images of the samples were taken in the same set up under cryogenic conditions using a Zeiss LD EC Epiplan Neofluar 100× objective with NA = 0.75 and standard rhodamine and fluorescein filter sets. The image pixel size is 9.8 nm for SXT and 148 nm for fluorescence microscopy.

### Registration of X-ray tomograms, 3D image reconstruction and alignment with fluorescence

Individual frames of tomograms with severe motion blur were manually removed before processing. For the alignment of tomograms containing gold beads as fiducial markers, the Bsoft software was used [100]. The tomograms lacking fiducial markers were aligned by an automatic alignment method developed within the CoCID project (https://cocid.eu/); separate manuscript in preparation) [101]. It is a feature-based method, using trackable features from the images as virtual markers for the alignment. The method uses a scale-free feature detection, such as in [102], to detect and initialize virtual markers. These virtual markers are expanded via feature tracking to provide observations of these features throughout the tilt series while using the uncertainty of the observations to accumulate an error estimate of the tracked path. The method utilizes similar methods to [103] for self-identifying and dealing with erroneous tracks, such as outliers and marker mixing. The algorithm successively tries to find suitable anchor points for the image with the most significant error of alignment and finishes when a preset criterion is met, after which image alignment can be solved in a weighted least-squares fashion. After the alignment, tomograms were binned by a factor of two. The reconstruction was carried out in Tomo3D using the filtered back projection algorithm [104]. For correlation of fluorescence and X-ray images, sum projections of portions of reconstructed 3D X-ray stacks were calculated to match the depth of field of the fluorescence microscope. Fluorescence images were deconvolved as described below prior to alignment with the reconstructed SXT sum projections.

### Fluorescence image preprocessing and cell segmentation using the circular Hough transform

Fluorescence images of yeast cells (BY4741, wild type; BY4742 for *ncr1∆* and *npc2∆*) using the green channel for droplets stained with BODIPY493/503 and the red channel for vacuoles stained with FM 4–64 were acquired on the Nikon and Leica wide-field microscopes (see above). ROIs are extracted using a protocol based on circular Hough transform. The first step is detecting edges using the Canny algorithm [105]. Then circular Hough transform is performed for an interval of radii corresponding to the possible cell dimensions. The best fitting circles across all intervals are found using a max projection. The max projection is blurred using a gaussian filter with σ=5, and peaks are finally found using local peak finding with a minimum distance set to the diameter of an average yeast cell for the acquisition. A ROI 100 × 100and 50 × 50pixel ROI is extracted around the peak, for Nikon and Leica acquisitions respectively. This protocol is fast but may detect circles outside of cells and does not account for low signal or high cell density, which can render the ROI unusable. ROIs can also be extracted using cell segmentation tools based on CNNs e.g [106–111], but may miss some cells and usually take much longer. A CNN classifier was trained to filter out the unusable ROIs (no cell, low signal, too crowded). In both classification CNNs, the ROIs are rescaled to 64 × 64using bicubic interpolation and their values are scaled between 0 and 1 by dividing by the acquisition’s saturation intensity (2047 and 7500 for Nikon and Leica, respectively).

### Deep learning model setup for vacuole phenotype classification

After generating the single yeast cell images, they must be annotated to be utilized for training and validation of the DYFNet model. 18362 images were manually annotated into two categories: one central vacuole corresponding to the fully fused state and multi-vesicular vacuole corresponding to the partially fused phenotype based as visible in the FM 4–64 vacuole staining. This step of the procedure is crucial, as wrongly annotated images can influence the output of the deep learning model. A third category was added for images which did not contain a single centered cell with sufficient signal (“rejected”). Two CNN models based on the architecture proposed by Zhang et al. 2020 were trained as part of DYFNet using the Nadam optimizer and binary cross-entropy. One model was trained on the fully and partially fused images and the other was trained on rejected and non-rejected images (those used to train the fully fused vs partially fused model). The models were implemented using Tensorflow. All images are normalized between 0 and 1 by dividing by the maximal intensity. Additionally, during training, we employ elastic deformation, random rotations and flipping to augment our data, greatly increasing generalization. Elastic deformations are implemented using Simard et al.’s algorithm. α and σ parameters are set to 60 and 15, respectively. A confusion matrix was generated from 8-fold cross-validation results to evaluate the performance of the DYFNet fully vs partially fused classifier. From the confusion matrix we can calculate the following performance metrics – Accuracy = 86.02%, sensitivity = 71.24%, specificity = 92.08%, PPV = 78.67%, NPV = 88.65%, MCC = 65.29%. The other classifier for rejecting images with unclear or missing cells had an accuracy of 89.85%, sensitivity of 87.38% and specificity of 91.77% with no clear bias towards the fully or partially fused cells. Sampling the misclassified images mostly revealed borderline images with lower signal. The two sets of classification models for rejecting unusable ROIs and for phenotype classification were applied on the full unlabeled dataset. Each set consists of the 8 models trained during cross-validation, and the final label is decided by the rounded mean output. To account for the expected error observed in the confusion matrices, we corrected the classification numbers based on the sensitivity and specificity of the models. Both the raw and the corrected predictions are plotted in Figure 2. To calculate pairwise P-values between the different conditions, we empirically assessed the probability of randomly observing a difference of means that was at least as high as the given value. Using a population size equal to the smaller of the compared pairs’ population, we sample two random distributions from the confusion matrix Over 100,000 iterations and observe the frequency of a difference of means equal to or higher than the given.

### Generation of training data for the segmentation model, preprocessing and data augmentation

Training data for the LipoSeg pipeline’s segmentation CNN was created by hand using ImageJ and 3D slicer. Many good segmentations of unseen data were added after post-processing (see below). Training data was verified using correlative fluorescent markers when available, and otherwise overlaid on the original data and evaluated by experts. In the final version of the training set, the cell membrane and droplet channel have 539 slices of training data across 28 tomograms. The vacuole channel has 56 slices across 21 tomograms. Each image in the Z-stack is rescaled to 512 × 512using bicubic interpolation. The original dimensions are stored in the program memory, such that the original pixel sizes can be recovered, after processing. The pre-processing consists of resizing to 512 × 512pixels and quantile normalization using a gaussian distribution with 0 mean and 1 standard deviation. This ensures consistent input for data that can have considerably different distributions. The procedure for this quantile normalization is described in the following pseudocode below:

1. **procedure** QuantileNormalize(*Img,Dist*) Algorithm 1

2. *Img*_*flat*_←Flatten (*Img*)

3. *Ranks*←ArgSort (*Img*_*flat*_)

4. Order←ArgSort (*Ranks*)

5. Imgflatnorm←Dist[order]

6. Imgnorm←Reshape(Iflatnorm,shape(Img))

7. **return** *Img*_*norm*_

Image augmentation includes 90-, 180-, and 270-degree rotations and elastic deformations. The rotations are multiples of 90, such that no information is lost to black bars or zoom. Elastic deformations are implemented using Simard et al.’s algorithm. α and σ parameters are set to 430 and 20, respectively, and were selected to get a meaningful distortion while maintaining recognizable features and patterns. A random seed is generated prior to deformation, such that all five images receive the same distortion.

### Architecture and training of the segmentation model

We decided to use a U-Net based architecture as already proven to be powerful for similar image processing tasks [59,60]. Other U-Net style architectures were tested but were dismissed to keep the complexity and capacity for overfitting down. The architecture is implemented using five encoder blocks, a bridge, five decoder blocks, and a single convolutional layer with sigmoid activation. Each encoder and corresponding decoder are connected through concatenation. Each successive encoder doubles the number of filters (starting with 32) and each decoder halves it again. Encoder blocks contain 2 sets of convolutions, batch normalization, and ReLU activation with the second set having half the number of filters in the convolutional layer. Each encoder is succeeded by a 2 × 2max pooling layer, halving the feature size in each dimension. The Decoder block is an encoder block preceded by an up-sampling layer, which doubles the feature map size in each dimension. All convolutional layers use zero padding to keep the image dimensions consistent and in powers of two. The input shape is 512 × 512x5. Five consecutive images are used to aide in consistency, reduce the effect of noise from individual slices and give the network some 3D context to combat the missing wedge problem.

A model is trained for each class. The training data is categorized according to the biological sample/tomogram and for each “epoch”, a random training pair is sampled from each tomogram. This reduces bias toward certain samples and phenotypes with more training data available. The Adam optimizer was selected based on an empirical review of optimizers. A compound loss function consisting of Dice and Top K cross entropy was selected, based on [112,113].

(1) $$\mathrm{DSC}=\frac{2\left|\hat{Y}\cup Y\right|}{\left|\hat{Y}\right|+\left|Y\right|}=\frac{2\mathrm{TP}}{\mathrm{TP}+\mathrm{FP}+\mathrm{TN}}$$

(2) $$\mathrm{Binary}\;\mathrm{Cross}\;\mathrm{Entropy}=-\frac{1}{N}\sum_{n=1}^{N}\left[y_{n}\log{\hat{y}}_{n}\;+\;\left(1-y_{n}\right)\log\left(1-{\hat{y}}_{n}\right)\right]$$

(3) $$\mathrm{Top}\;\mathrm{KBCE}=-\frac{1}{N}\sum_{n=1}^{N}\phi_{n}*\left[y_{n}\log{\hat{y}}_{n}\;+\;\left(1-y_{n}\right)\log\left(1-{\hat{y}}_{n}\right)\right]$$

where $\phi_{n}=\left\{\begin{array}{c} 1\;\;\;\;\;\mathrm{if}\;n\;\mathrm{is}\;\mathrm{in}\;\mathrm{the}\;\mathrm{top}\;k\;\mathrm{biggest}\;\mathrm{losses}\\ 0\;\;\;\;\;\mathrm{otherwise} \end{array}\right.$

Models were trained iteratively on each channel, as more training data was created. The vacuole channel was trained using the cell membrane channel as initial model weights.

### Postprocessing and analysis of segmented 3D X-ray image stacks

Outlier removal removes artifacts outside of the cell membrane. This is accomplished by thresholding the mean Z-projection of the cell membrane prediction stack and expanding it using binary dilation. This mask is then applied to the cell membrane channel. The similar masking option uses the post-processed cell membrane segmentation as a mask for other channels with the masking option enabled. This prevents misclassifications of cellular components outside of the cell membrane (e.g., fiducial markers which may resemble LDs). The averaging option is implemented using a 3D gaussian filter which increases consistency in areas with low prediction confidence and slices causing unexpected drops resulting in false negatives. Hole filling is implemented using SciPy’s *binary_fill_holes* [114]. The implementation fills the holes by dilating the complementary of the input. Hole filling is conducted after instance segmentation, such that gaps between cells and cellular components are not wrongly filled. Hole filling can aid in predictions where smaller sections fall below the classification threshold and generate holes in the membrane or vacuole. The convex constraint is implemented by reducing point clouds to convex hulls [115]. This can help in cases where the segmentation is known to be convex but yields a concave shape. After mesh generation using marching cubes, meshes may be smoothed using Laplacian smoothing.

For the analysis, 28 *S. cerevisiae* yeast cell tomograms were segmented using cell membrane, vacuole, and droplet channels. 14 WT, 6 Ncr1 and 8 Npc2. The segmentations were sorted into those where a vacuole could be identified and segmented and those that could not. For each droplet in all segmentations, volume, distance to nearest vacuole, whether it was consumed by a vacuole and the sample type were recorded. Distance was estimated by raytracing between centers of mass. LDs were considered consumed if their center of mass was enclosed by the nearest vacuole’s surface. The distribution of droplet volumes between wild type and Ncr1 were compared. Additionally, in all samples with well-segmented vacuoles, the distributions of volumes of LDs inside and outside vacuoles were plotted against each other using gaussian kernel density estimation and p-values were acquired using method Mann-Whitney U-test and the Kolmogorov – Smirnov test. Similar comparisons were made under other conditions but did not yield p-values under 0.05. Image simulations were carried out in ImageJ, while tomographic reconstruction of the simulated yeast phantom images was implemented in the skimage Python library skimage.transform.

## Acknowledgments

DW acknowledges funding from the Villum Foundation (grant no. 35865) and from the Danish Research Council (grant ID: 2032–00139B). Image acquisition was performed at the Danish Molecular Biomedical Imaging Center (DaMBIC, University of Southern Denmark), supported by the Novo Nordisk Foundation (NNF) (grant agreement number NNF18SA0032928).

## Supplementary Material

Supplemental Material

Supplemental Material

## Data Availability

The code for the classification CNN and for the segmentation pipeline is available at https://github.com/Wuestner-Lab/DYFNet and https://github.com/Wuestner-Lab/LipoSeg, respectively. Selected examples of 3D reconstructions from X-ray tomograms and all fluorescence images of the trainings set are availability at https://zenodo.org/record/8070003 with this link additionally provided at the DYFNet GitHub page.
